# The effects of baicalin in depression: preclinical evidence construction based on meta-analysis

**DOI:** 10.3389/fphar.2024.1425094

**Published:** 2024-07-24

**Authors:** Dan Wang, Yu-Meng Ren, Yi-Xuan Guo, Zhi-Qi Zhang, He- Sui, Hai-Yan Zhang

**Affiliations:** ^1^ College of Traditional Chinese Medicine, Shandong University of Traditional Chinese Medicine, Jinan, China; ^2^ School of Pharmacy, Shandong University of Traditional Chinese Medicine, Jinan, Shandong, China; ^3^ School of Acupuncture-Moxibustion and Tuina, Shandong University of Traditional Chinese Medicine, Jinan, China; ^4^ Research Institute of Acupuncture and Moxibustion, Shandong University of Traditional Chinese Medicine, Jinan, China

**Keywords:** baicalin, flavonoids, meta-analysis, depression diseases, review

## Abstract

**Background:**

Depression manifests as a mental disorder characterized by a low mood, suicidal tendencies, disturbances in sleep-wake cycles, psychomotor agitation, and pronounced feelings of hopelessness and anhedonia. Baicalin, a natural flavonoid compound, shows significant promise in alleviating depressive symptoms in animals. This study aims to assess the impact of baicalin on experimental models of depression.

**Methods:**

A systematic search of electronic databases was conducted using the search terms “baicalin” AND “depression” OR “depressed” OR “anti-depression”. Preclinical animal models representing experimental depression were included in the analysis. The risk of bias in the included studies was evaluated using the CAMARADES tools.

**Results:**

Baicalin significantly increased sucrose preference test (SPT) [SMD= 21.31, 95%CI (16.32, 26.31), *P* < 0.00001]. mThe tail suspension test (TST) duration significantly decreased in the baicalin group compared to the model group [SMD = −39.3, 95%CI (−49.71, −28.89), *P* < 0.0001]. Furthermore, baicalin reduced immobility time in rats subjected to the forced swim test (FST) [SMD = −39.73, 95%CI (−48.77, −30.69) *P* < 0.0001]. Compared to the model group, baicalin treatment also significantly increased the frequency of crossings in the open field test (OFT) [SMD = 32.44, 95%CI (17.74, 47.13), *P* < 0.00001].

**Conclusion:**

Baicalin significantly improves the manifestations of depressive symptoms. The effect of baicalin against depression is exerted through its anti-inflammatory actions, inhibition of oxidative stress, regulation of the HPA axis, and restoration of neuroplasticity. Future studies will be needed to further explore how these promising preclinical findings can be translated into clinical treatment for depression.

**Systematic Review Registration:**

https://www.crd.york.ac.uk/PROSPERO/, identifier CRD42023472181.

## 1 Introduction

Depression is classified as a mental disorder characterized primarily by persistent low mood, diminished interest or pleasure in activities, reduced initiative, and lack of energy (Marwaha et al., 2023). The research indicates that as a consequence of the global COVID-19 pandemic in 2020, there was a 27.6% increase in the number of patients with severe depressive disorder worldwide (Myles et al., 2023). According to a report by the World Health Organization (WHO), as of March 2023, 280 million people worldwide suffer from depression, and more than 700,000 people die each year due to impulsive behaviors related to depression (Jing and Ding, 2024). Currently, depression stands as one of the leading causes of disability and contributes significantly to the overall global disease burden (Zheng S. et al., 2024). It imposes substantial psychological and financial burden on patients and their families, making it an urgent public health issue that requires immediate attention (Haghish et al., 2024).

To date, the etiological mechanisms of depression remain incompletely understood, potentially involving factors such as inflammation, neurotransmitters, neuroplasticity and neuroregeneration, the hypothalamic-pituitary-adrenal (HPA) axis, as well as genetic and environmental influences (Peters et al., 2024). Recent studies have shown that chronic psychosocial stress leads to persistent activation of the HPA axis, subsequently triggering the release of glucocorticoids, which is closely associated with the onset of depression (Liu et al., 2024). Patients with depression often exhibit dysfunction of the HPA axis, accompanied by elevated levels of circulating glucocorticoids (Qin et al., 2024). However, elevated glucocorticoid levels further impair central nervous system function, leading to reduced hippocampal neurogenesis and inducing neuronal apoptosis (Qin et al., 2024). The onset of depression is also associated with reduced expression of neurotrophic factors (Zhou et al., 2024). Inhibition of the expression of brain-derived neurotrophic factor (BDNF) and nerve growth factor (NGF) in the peripheral nervous system is related to abnormal neural function in the brains of patients with depression (Luo et al., 2023). Abnormal oxidative stress and inflammation in patients with depression impair neuroplasticity by inhibiting the expression of BDNF and NGF (Luo et al., 2023).

Currently, the primary treatments for depression include cognitive behavioral therapy, transcranial direct current stimulation, and pharmacotherapy (Kong et al., 2024). Most antidepressants are developed based on the principle of increasing the concentration and utilization of monoamine neurotransmitters in the synaptic clefts of central neurons (Colla et al., 2024). For instance, the first generation of antidepressants primarily consists of tricyclic antidepressants, which work by increasing the transmission of monoamine neurotransmitters, and monoamine oxidase inhibitors (MAOIs), which primarily act by inhibiting the degradation of these neurotransmitters (Kim and Amsterdam, 2023). Modern antidepressants commonly used today primarily function by inhibiting the reuptake of serotonin (5-HT), norepinephrine (NE), and dopamine (DA) (Veraart et al., 2022). Examples include selective serotonin reuptake inhibitors (SSRIs), serotonin-norepinephrine reuptake inhibitors (SNRIs), and norepinephrine-dopamine reuptake inhibitors (NDRIs) (Hovorka et al., 2022). These antidepressants often come with side effects, and their overall efficacy ranges from 50% to 75% (Laux, 2022). Therefore, finding safe and effective medications for the treatment of depression has become a major focus of research.

Baicalin is a flavonoid compound isolated from the roots of Scutellaria baicalensis, which possesses various biological activities including antibacterial, diuretic, anti-inflammatory, and antioxidant effects (Cho et al., 2024). Research has shown that it possesses strong neuroactive and neuroprotective properties (Zheng et al., 2024b). It can effectively alleviate nerve damage caused by glucose-oxygen deprivation, inhibit the overactivation of NMDAR glutamate receptors, counteract central inflammatory responses and oxidative stress, and promote neuronal cell differentiation (Zhong et al., 2019). In addition, it improves nerve damage induced by scopolamine and seizure-like symptoms in rats (Cintra et al., 2024). Recent studies have found that baicalin can effectively alleviate depressive symptoms caused by chronic stress in rats, demonstrating strong antidepressant activity (Fan et al., 2020; Chalermwongkul et al., 2023; Hu et al., 2023).

At present, accumulating pharmacological evidence suggests that baicalin may hold significant potential in the treatment of depression (X. Jin et al., 2023; Ma et al., 2023). However, research on the use of baicalin for treating depression has not been comprehensively evaluated or summarized. This study provides a rigorous and comprehensive systematic review and meta-analysis of the literature concerning the use of baicalin in treating depression in animal models. It explores various behavioral changes and potential mechanisms, aiming to provide evidence for clinical practice.

## 2 Materials and methods

Our research adhered to the established procedures specified in the Cochrane Handbook for Meta-Analyses and Systematic Reviews. For reporting, adherence to the PRISMA requirements was guaranteed. The study protocol received registration at Prospero (CRD42023472181).

### 2.1 Search strategies

A comprehensive search was conducted in PubMed, Embase, PsycINFO, Web of Science, China National Knowledge Infrastructure (CNKI) database, and Cochrane Library for related publications from their inception until 3 November 2024. Additionally, reference lists of relevant studies were also screened, and Clinical Trials. gov was searched for further pertinent literature. The search keywords were formulated as follows: (baicalin) And (“Depression” OR “Depressed” OR “Anti-depression”).

### 2.2 Inclusion and exclusion criteria

Studies were included if they met the following criteria: 1) Conducted *in vivo* studies involving animal subjects; 2) Utilized an animal model of depressive disorder; 3) Administered treatment with baicalin. Exclusion criteria were as follows: 1) Studies of other types, such as *in vitro* studies, case reports, clinical trials, reviews, abstracts, or comments; 2) Combination treatments with other compounds or herbal products; 3) Not using a depressive disorder model; 4) Studies with insufficient data.

### 2.3 Literature screening and data extraction

During the literature screening phase, two independent researchers reviewed the studies based on predetermined inclusion and exclusion criteria. In cases of discrepancies, a third researcher arbitrated the final decision after discussion. The following details were extracted for each study: 1) Year of publication and first author’s name; 2) Animal characteristics, including species, number, sex, weight; 3) Methodology for establishing the depressive disorder model; 4) Intervention specifics, including dosage and administration route; 5) Primary outcome measures and intergroup differences.

### 2.4 Quality assessment of included studies

In this study, two researchers conducted a systematic review of the included studies using the Collaborative Approach to Meta-Analysis and Review of Animal Data from Experimental Studies (CAMARADES) risk of bias tool. The evaluation criteria comprised: 1) Peer-reviewed publication; 2) Temperature control; 3) Random allocation to experimental groups; 4) Blinded induction of depressive disorder; 5) Blinded assessment of behavioral outcomes; 6) Use of anesthetics without significant intrinsic neuroprotective activity; 7) Calculation of sample size to achieve adequate statistical power; 8) Utilization of appropriate animal models without relevant comorbidities (e.g., aged, diabetic, or hypertensive animals); 9) Adherence to animal welfare regulations; 10) Disclosure of potential conflicts of interest. Any disagreements between the two researchers were resolved through discussion with a third researcher.

### 2.5 Statistical analysis

Statistical analysis of the data was conducted using Review Manager 5.3 (Cochrane, London, UK), and sensitivity analysis was performed using STATA 15 (Stata Corporation, College Station, TX, United States). This study utilizes relative risk (RR) and 95% confidence interval (CI) as indicators for dichotomous variables, while the standardized mean difference (SMD) and 95% CI are used for continuous variables. The Q test was used to analyze study heterogeneity, with the I^2^ test aiding in determining the extent of heterogeneity. If I^2^ ≤ 50%, it indicates small heterogeneity between studies, and the fixed effects model is applied. Conversely, if I^2^ ≥ 50%, it suggests large heterogeneity between studies, and a random effects model is utilized. Publication bias was analyzed qualitatively using funnel plots and statistically using Egger’s test.

## 3 Results

### 3.1 Research screening

**FIGURE 1:**
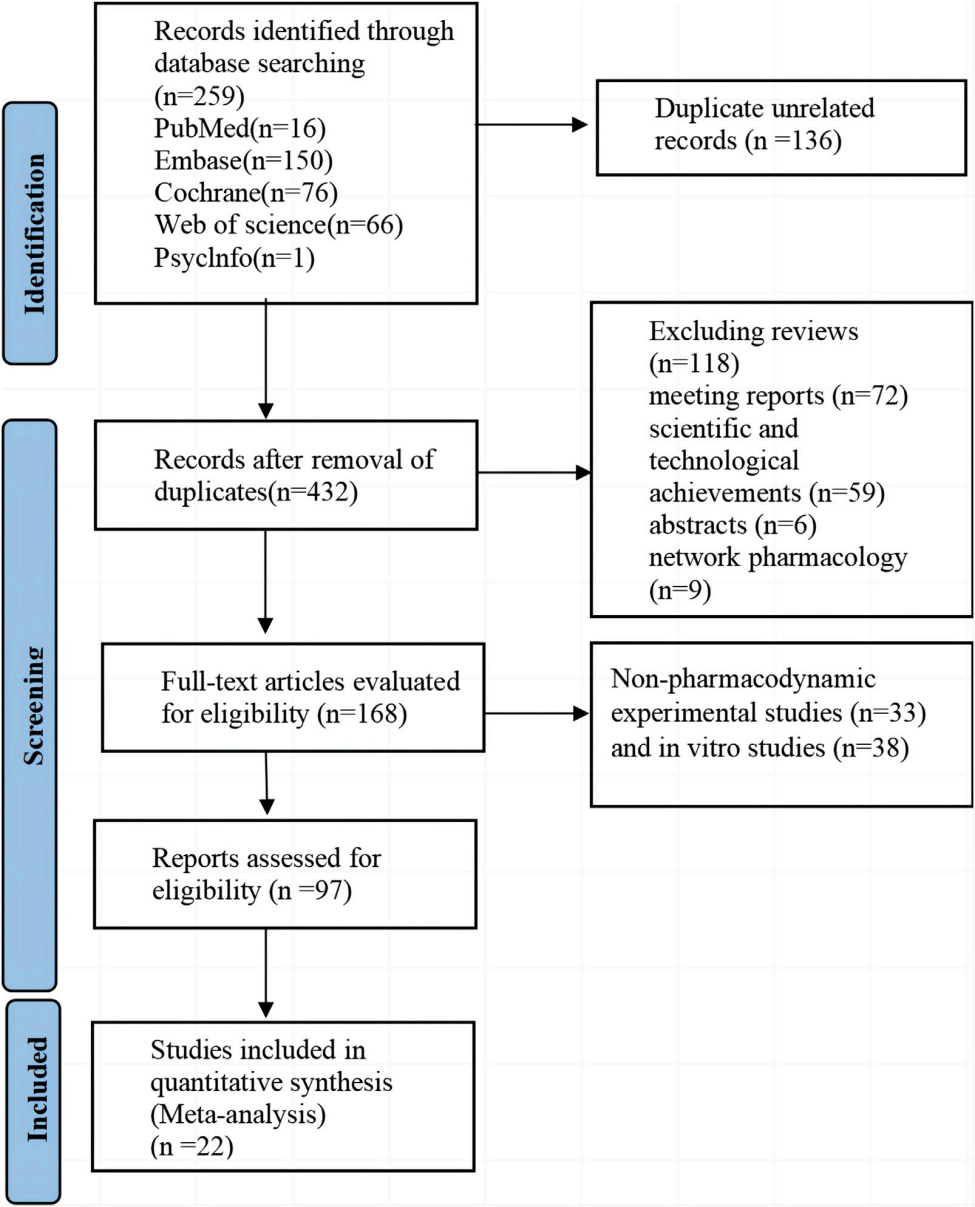
Literature retrieval flow chart.

According to the preset retrieval strategy, a total of 568 articles were retrieved. After the exclusion of unrelated articles, 432 articles remained. Further exclusions included reviews (n = 118), meeting reports (n = 72), scientific and technological achievements (n = 59), abstracts (n = 6), and network pharmacology studies (n = 9). Thus, 168 studies were included in the full manuscript screening. Non-pharmacodynamic experimental studies (n = 33) and *in vitro* studies (n = 38) were excluded by reading titles and abstracts. Finally, 22 articles were included for comprehensive analysis after excluding articles that did not meet the outcome criteria (n = 26) and articles that could not provide data (n = 49). The detailed literature search and screening process is illustrated in Figure 1.

### 3.2 Characteristics of included studies

The basic characteristics of the 22 studies are shown in Table 1. This meta-analysis included a total of 1,050 animals, with 700 in the model group and 350 in the baicalin group. The model group consisted of four different animal models of depression: chronic unpredictable mild stress (CUMS), corticosterone (CORT), olfactory bulbectomy (OBX), and lipopolysaccharide (LPS). The model group received oral treatments with fluoxetine, fasudil, or amitriptyline. The baicalin group was treated only with baicalin at doses ranging from 3.35 to 160 mg/kg for a duration of 1–5 weeks.

**TABLE 1:** Characteristics of included studies.

Studies	Species (sex, n)	Model	Weigh (g)	Experimental group	Control group	Treatment time/day	Outcome
Shen et al. (2023)	ICR (Male, 50)	Cort	18–22	Baicalin (10 \20 mg/kg)	Fluoxetine (20 mg/kg)	21	②③
Wang et al. (2016)	SD (Male,35)	CUMS	230 ± 18	Baicalin (20 \40 mg/kg)	Fluoxetine (10 mg/kg)	35	②③
Liu (2019)	ICR (Male,60)	CUMS	30 ± 3	Baicalin (4\8\12 mg/kg)	Fluoxetine (5.2 mg/kg)	21	②③
Zhao et al. (2020)	C57BL/6J (Female,50)	Cort	18–22	Baicalin (30 \60 mg/kg)	Escitalopram (10 mg/kg)	42	①②③④
Shen et al. (2016)	SD (Male,48)	LPS	300 ± 20	Baicalin (146.4 mg/kg)	Fluoxetine (7 mg/kg)	7	②③
Li et al. (2015)	Wistar (Male)	CUMS	180–220	Baicalin (10 \20\40 mg/kg)	Fluoxetine (7 mg/kg)	35	②③
Li et al. (2013)	ICR (Male,78)	Cort	18–22	Baicalin (10 \20 mg/kg)	Fluoxetine (20 mg/kg)	42	②③
Zhang et al. (2019b)	C57BL/6J (Male,36)	Cort	18–22	Baicalin (40 80\160 mg/kg)	Fluoxetine (18 mg/kg)	56	②④
Liu et al. (2017)	Male,60	CUMS	180–220	Baicalin (20 \40 mg/kg)	Fluoxetine (10 mg/kg)	42	②③
Guo et al. (2019)	C57BL/6 (Male)	Cort	—	Baicalin (3.35 \6.7 mg/kg)	Fluoxetine (18 mg/kg)	30	④
Zhang et al. (2018a)	SD (Male,60)	CUMS	180–220	Baicalin (20 \40 mg/kg)	Fluoxetine (10 mg/kg)	42	②③
Zhang et al. (2019a)	SD (Male,60)	CUMS	190–220	Baicalin (500 \1000 mg/kg)	Fluoxetine (10 mg/kg)	42	②③
Deng et al. (2019)	ICR (Male)	CUMS	20–22	Baicalin (30 \60 mg/kg)	Fasudil (20 mg/kg)	49	②③④
Gao et al. (2018)	ICR (Male,60)	CUMS	18–22	Baicalin (30 \60 mg/kg)	Fluoxetine (20 mg/kg)	35	②③④
Liu et al. (2019)	ICR (60)	CUMS	18–22	Baicalin (10 \20 mg/kg)	Fluoxetine (20 mg/kg)	70	②③④
Lu et al. (2019)	Male,48	CUMS	20–25	Baicalin (25 \50\100 mg/kg)	Fluoxetine (10 mg/kg)	7	②③
Yu et al. (2019)	SD (Male,72)	OBX	180–220	Baicalin (20 \40 mg/kg)	Amitriptyline (10 mg/kg)	14	②③
Zhang et al. (2016)	C57BL/6 (Male,90)	Cort	18–22	Baicalin (40 \80\160 mg/kg)	Fluoxetine (18 mg/kg)	56	②④
Zhang et al. (2018b)	ICR (Male,48)	CUMS	23–26	Baicalin (60 mg/kg)	Fluoxetine (15 mg/kg)	42	③④
Zhong et al. (2019)	C57BL/6 (Male,40)	CUMS	—	Baicalin (25 \50 mg/kg)	Fluoxetine (20 mg/kg)	42	②③④
Jia et al. (2021)	ICR (Male,50)	CUMS	24 ± 2	Baicalin (25\50 mg/kg)	Fluoxetine (10 mg/kg)	63	③④
Xiao et al. (2021)	ICR (Male,45)	CUMS	20–25	Baicalin (50 \100 mg/kg)	Fluoxetine (10 mg/kg)	42	②③

The animal species involved are C57BL/6 (130/1,050) accounting for 12.38%, C57BL/6J (86/1,050) accounting for 8.19%, Institute of Cancer Research (ICR) (451/1,050) accounting for 42.95%, and Sprague Dawley (SD) (275/1,050) accounting for 26.19% (Figure 3A). The proportion of male and female animals was 89.52% (940/1,050) and 4.76% (50/1,050), respectively (Figure 3B). Furthermore, 31.90% (335/1,050) of animals were rats, and 68.10% (715/1,050) of animals were mice (Figure 3C). The body weight of rats and mice ranged from 18g to 320 g. The distribution is as follows: 63.64% (14/22) CUMS, 27.27% (6/22) CORT, 4.55% (1/22) LPS, and 4.55% (1/22) OBX (Figure 3D). In terms of quality assessment scores, 18% (4/22) of the studies had seven points, 27% (6/22) of the studies had eight points, and 55% (12/22) of the studies had nine points (Figure 3E).

### 3.3 Quality evaluation

**FIGURE 2:**
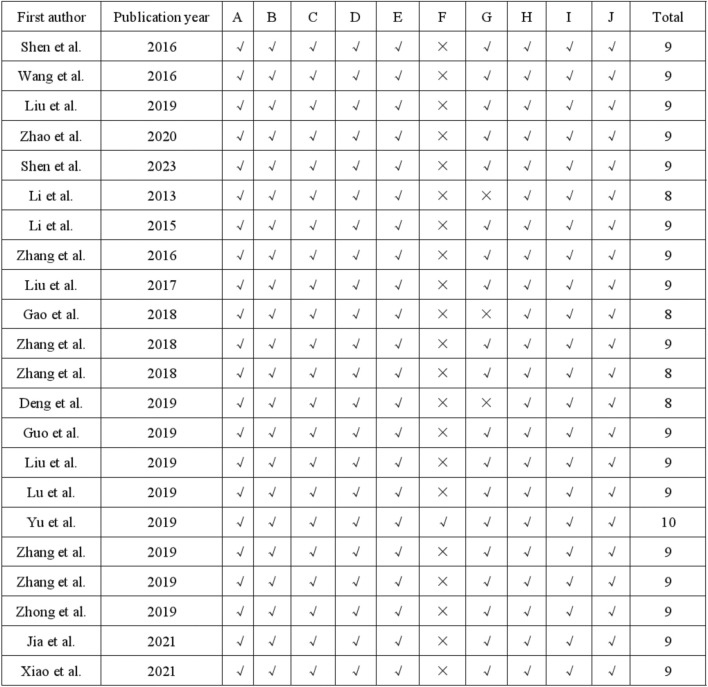
Risk of bias and quality assessment scores in each study.

**FIGURE 3:**
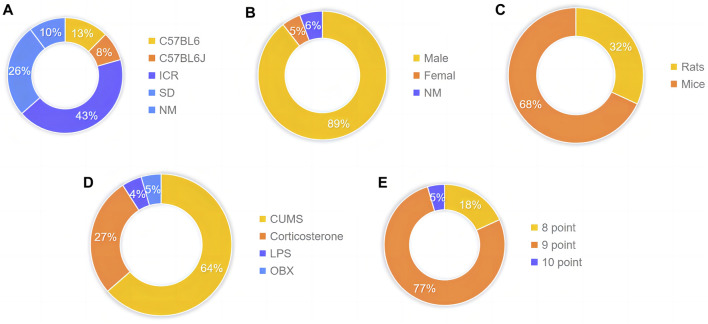
Characteristics of eligible studies. **(A)** Animal species **(B)** gender proportion **(C)** Type classification **(D)** Experimental model classification **(E)** Quality assessment scores.

The quality assessment of the 22 studies was conducted using the 10-item CAMARADES checklist, with scores ranging from 8/10 to 10/10. Specifically, all studies utilized appropriate animal models of depression and calculated sample sizes. Additionally, all 22 studies mentioned random allocation and concealed distribution. Twenty-one studies avoided the use of neurotoxic or protective anesthetics. Furthermore, all studies demonstrated compliance with animal welfare principles and declared no conflicts of interest. The specific risk of bias items is presented in Figures 2, 3E.

### 3.4 Outcome measures

The outcome measures in the 22 studies encompassed assessments of behavioral changes, pathological alterations, and histological analyses. Behavioral changes were evaluated using the sucrose preference test (SPT), tail suspension test (TST), forced swim test (FST), and open field test (OFT). Notably, the histological analyses focused on the CA1, CA3, and dentate gyrus regions of the hippocampus, as well as the prefrontal cortex and hypothalamus, due to their critical roles in mood regulation and stress response. These analyses further elucidated the specific mechanisms underlying the antidepressant effects of baicalin.

### 3.5 Effects of baicalin on depression

#### 3.5.1 Effects of baicalin on depression by SPT analysis

**FIGURE 4:**
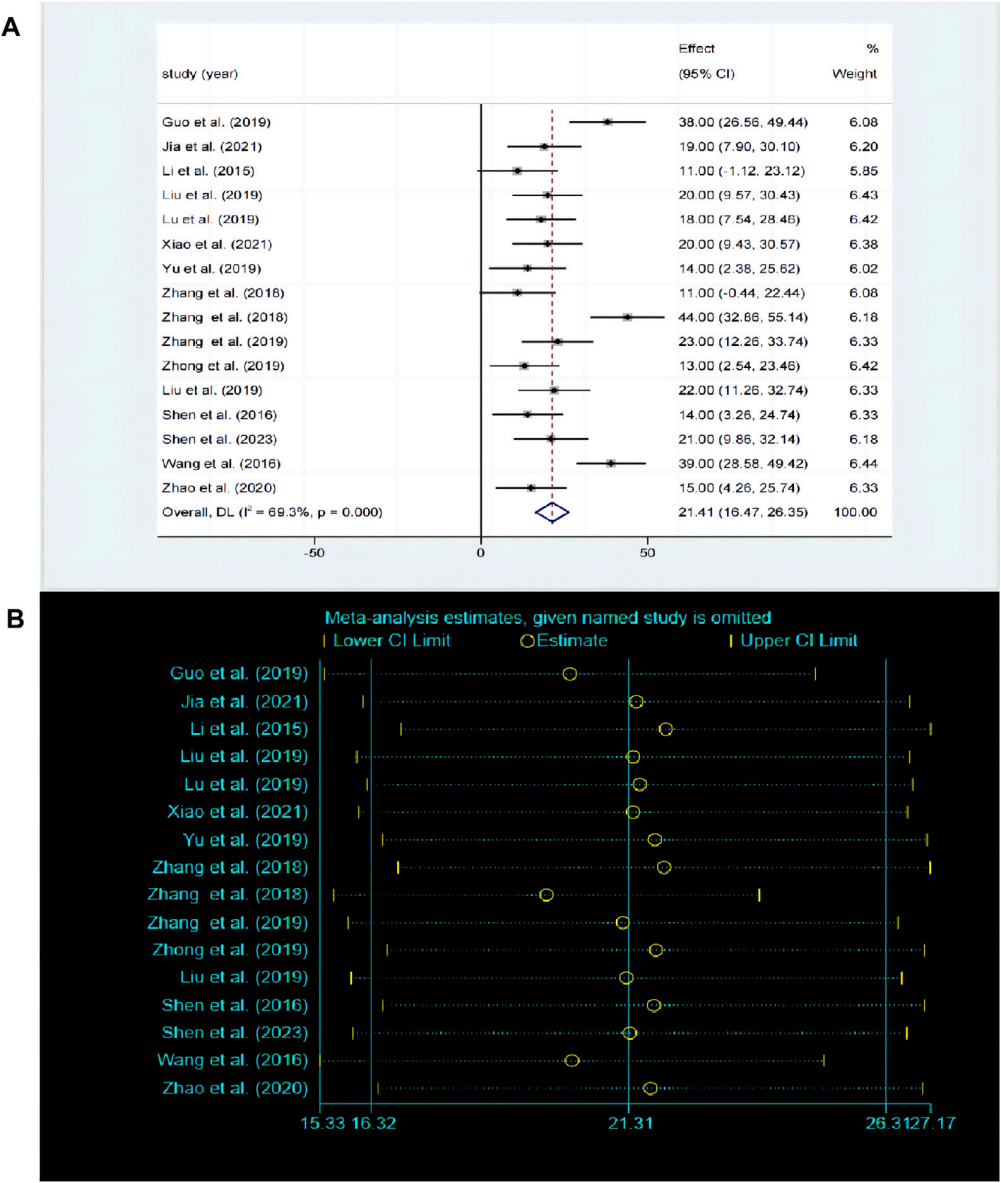
**(A)** Forest map and **(B)** sensitivity analysis of SPT.

The SPT is widely recognized as a prevalent method for assessing anhedonia. A total of 17 studies compared the differences in SPT before and after baicalin treatment. Due to significant heterogeneity between studies (*P* < 0.00001, I^2^ = 69.3%), the random-effects model was adopted. Compared with model group, baicalin could significantly increase the preference for sucrose [SMD = 21.31, 95%CI (16.32, 26.31), *P* < 0.00001] (Figure 4A). The sensitivity analysis indicated that the findings were robust and consistent (Figure 4B).

#### 3.5.2 Effects of baicalin on depression by TST analysis

**FIGURE 5:**
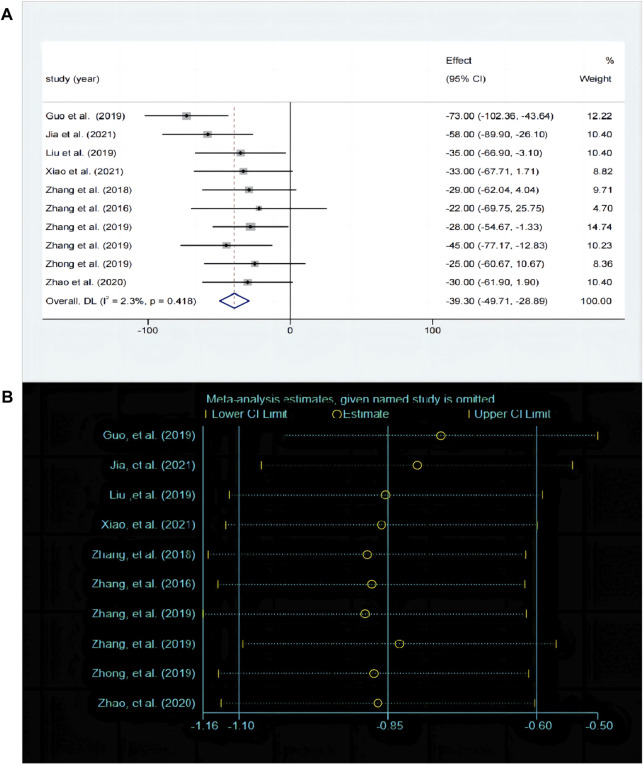
**(A)** Forest map and **(B)** sensitivity analysis of TST.

The TST is the most commonly used behavioral despair model experiment and serves as a classic method for evaluating antidepressant drugs. A total of 10 studies compared the differences in TST before and after baicalin treatment. The fixed effects model was employed (*P* < 0.00001, I^2^ = 0.42%). Compared with model group, the immobility time was significantly decreased in the baicalin treatment group [SMD = −39.3, 95%CI (−49.71, −28.89), *P* < 0.0001] (Figure 5A). The sensitivity analysis indicated that the findings were robust and consistent (Figure 5B).

#### 3.5.3 Effects of baicalin on depression by FST analysis

**FIGURE 6:**
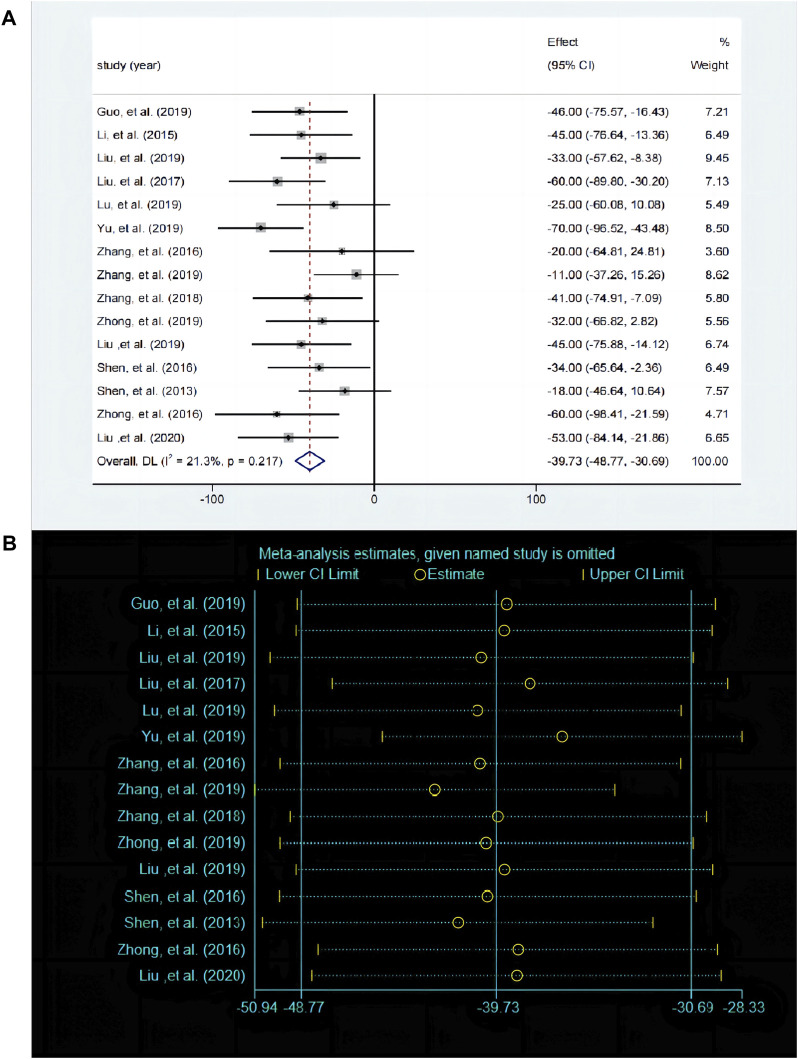
**(A)** Forest map and **(B)** sensitivity analysis of FST.

The FST is used to evaluate despair behavior in mice or rats and serves as a key indicator for assessing depressive-like behavior. A total of 15 studies compared the differences in FST before and after baicalin treatment. The fixed effects model was employed (*P* < 0.00001, I^2^ = 18.8%). Compared with the control group, baicalin treatment also reduced the inactivity time [SMD = −39.86, 95%CI (−48.9, −30.82), *P* < 0.0001], suggesting that it has an antidepressant like behavioral effect (Figure 6A). The sensitivity analysis revealed that the results were robust and consistent (Figure 6B).

#### 3.5.4 Effects of baicalin on depression by OFT analysis

**FIGURE 7:**
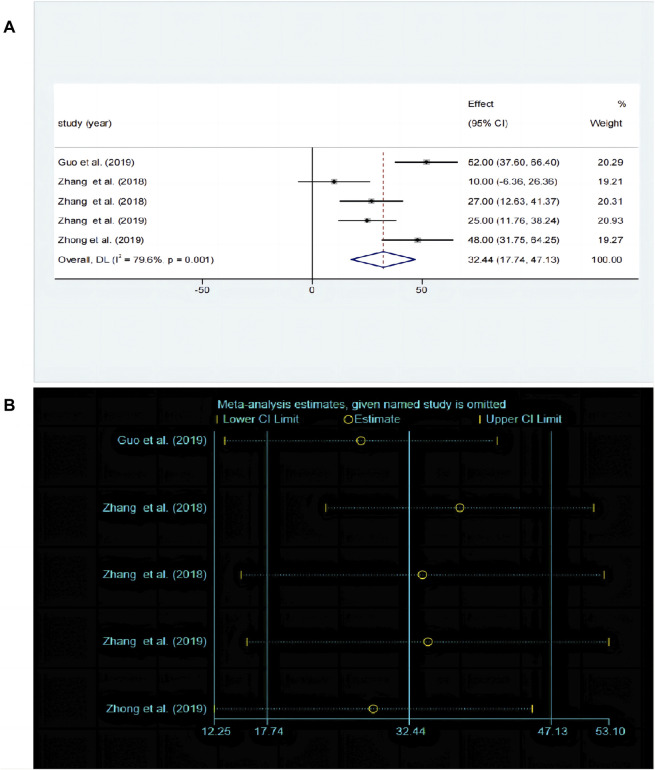
**(A)** Forest map and **(B)** sensitivity analysis of OFT.

The OFT is a widely used experimental paradigm based on observing the behavioral characteristics, exploratory behavior, and anxiety levels of animals in unfamiliar environments. A total of five studies (Li et al., 2013; Jiduo et al., 2016; Yue et al., 2016; Lu et al., 2019; Yongyong et al., 2019) compared the differences in OFT before and after baicalin treatment. Due to significant heterogeneity between studies (*P* < 0.00001, I^2^ = 79.6%), the random-effects model was adopted. Baicalin treatment significantly improved the frequency of crossing compared to the model group [SMD = 32.44, 95%CI (17.74–47.13), *P* < 0.00001] as illustrated in Figure 7A. The sensitivity analysis revealed that the results were robust and consistent (Figure 7B).

### 3.6 Publishing Bias

**FIGURE 8:**
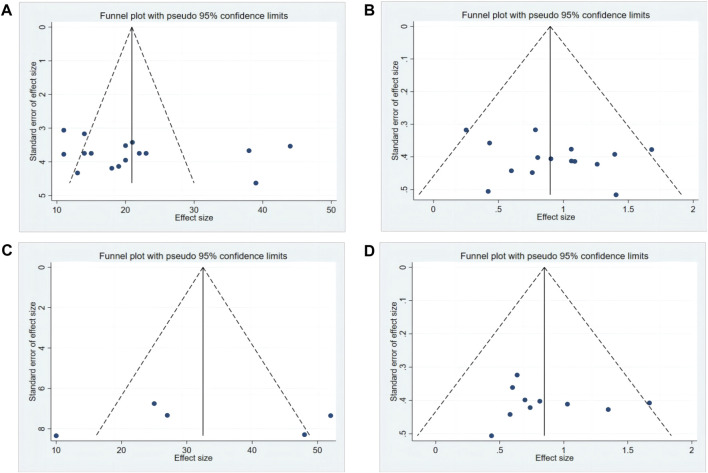
Publication bias of baicalin. **(A)** SPT **(B)** TST **(C)** FST **(D)** OFT.

Publication bias regarding the changes in SPT, FST, OFT, and TST was evaluated using a funnel plot. The included studies were evenly and symmetrically distributed on both sides of the axis, under the cover of the triangle in the funnel plots, suggesting a minimal risk of publication bias. This was additionally validated by the outcomes of Egger’s test (*P* > 0.05) (Figure 8).

### 3.7 Subgroup analysis

To investigate the impact of different factors on outcome measures, we conducted a subgroup analysis of the baicalin group based on baicalin dosage, animal species, and depression animal models (Table 2). When conducting a subgroup analysis, it is essential to include at least two articles; a single article will not be subjected to subgroup analysis.

**TABLE 2:** Subgroup analysis outcomes after baicalin intervention.

Outcome	No.of study	Variable	Heterogeneity		Effect size (95%CI)	*P*
			I^2^ (%)	*P*		
SPT	Animal model					
	8	CUMS	0.01	0.851	24.29 (17.72, 30.87)	0.0001
	6	Cort	4.3	0.382	13.00 (9.07, 16.93)	0.0001
	Daily dose (mg/kg)					
	3	≤20	0.01	0.828	36.70 (19.93, 53.47)	0.0001
	2	20–40	0.01	0.677	66.77 (44.77, 88.77)	0.0001
	6	40–60	0.01	0.891	47.11 (34.00, 60.22)	0.0001
	4	60–100	0.01	0.939	17.21 (1.04, 33.38)	0.0001
	Animal species					
	3	Rat	0.01	0.637	37.40 (28.27, 46.53)	0.0001
	12	mouse	71.9	0.029	48.98 (15.18, 82.78)	0.0001
TST	Animal model					
	7	CUMS	10.4	0.350	−43.99 (−57.3, −30.66)	0.0001
	3	Cort	0.01	0.965	−27.78 (−47.06, −8.50)	0.005
	Daily dose (mg/kg)					
	2	20–40	0.01	0.287	−29.00 (−62.50, −4.50)	0.09
	6	40–60	19.4	0.287	−45.49 (−60.44, −30.54)	0.001
	3	60–100	0.01	0.938	−28.57 (−48.43, −8.71)	0.005
	Animal species					
	9	rat	3.7	0.404	−29.00 (−62.50, −4.50)	0.0001
FST	Animal model					
	8	CUMS	0.01	0.851	−44.78 (−56.48, −33.09)	0.0001
	5	Cort	4.3	0.382	−30.16 (−44.18, −16.14)	0.0001
	Daily dose (mg/kg)					
	3	≤20	0.01	0.828	−36.70 (−53.47, −19.93)	0.0001
	2	20–40	0.01	0.677	−66.77 (−88.77, −44.77)	0.0001
	6	40–60	0.01	0.891	−47.11 (−60.22, −34.00)	0.0001
	4	60–100	0.01	0.939	−17.21 (−33.38, −1.04)	0.037
	Animal species					
	3	Rat	71.9	0.029	−48.98 (−82.78, −15.18)	0.005
	12	mouse	0.01	0.637	−37.40 (−46.53, −28.27)	0.0001
OFT	Animal model					
	5	CUMS	79.6	0.001	32.44 (17.74, 47.13)	0.0001
	Daily dose (mg/kg)					
	2	20–40	0.01	0.001	10.00 (6.36, 26.36)	0.231
	3	40–60	76.6	0.014	41.33 (23.94, 58.73)	0.0001
	2	60–100	0.01	0.018	27.00 (12.63, 41.37)	0.0001
	Animal species					
	2	Rat	76.6	0.014	18.97 (2.33, 35.60)	0.025
	3	mouse	57.3	0.126	41.33 (23.94, 58.73)	0.0001

#### 3.7.1 SPT

The animals were divided into two groups based on the animal model of depression: CUMS and CORT. The results demonstrated that after baicalin treatment, both the CUMS group [SMD = 24.29, 95%CI (17.72, 30.87), *P* < 0.00001] and the CORT group [SMD = 30.16, 95%CI (16.14, 44.18), *P* < 0.00001] showed a significantly increased preference for sucrose compared to the model group. These findings indicate that the improvement of SPT by baicalin is not affected by different animal models of depression. The baicalin intervention doses were divided into four groups: ≤20 mg/kg, 20–40 mg/kg, 40–60 mg/kg, and 60–100 mg/kg. Compared to the model group, sucrose intake in the baicalin groups significantly increased under the doses of ≤20 mg/kg [SMD = 36.70, 95%CI (19.93–53.47), *P* < 0.00001], 20–40 mg/kg [SMD = 66.77, 95%CI (44.77, 88.77), *P* < 0.00001], 40–60 mg/kg [SMD = 47.11, 95%CI (34.00, 60.22), *P* < 0.00001], and 60–100 mg/kg [SMD = 17.21, 95%CI (1.04, 33.38), *P* < 0.00001]. The results indicate that the improvement effect of baicalin on the SPT in depression animal models is not affected by the dosage of baicalin. The animals were divided into two groups according to their species: rats and mouse. Compared with the model group, the baicalin showed a significant improvement in sucrose preference in both rat [SMD = 37.40, 95%CI (28.27, 46.53), *P* < 0.00001] and mouse [SMD = 48.98, 95%CI (15.18, 82.78), *P* < 0.00001], suggesting that the effect of baicalin on improving SPT is not affected by the animal species. Therefore, the improvement effect of baicalin on SPT is not influenced by different depression models, baicalin doses, or animal species.

#### 3.7.2 TST

The animals were divided into two groups based on the animal model of depression: CUMS and CORT. The results indicate that baicalin treatment significantly reduced the immobility time compared with the model group in both the CUMS group [SMD = −43.99, 95%CI (−57.31, −30.66), *P* < 0.00001] and the CORT group [SMD = −27.78, 95%CI (−47.06, −8.50), *P* < 0.00001]. These findings indicate that the improvement of TST by baicalin is not affected by different animal models of depression. The baicalin intervention doses were also divided into three groups: 20–40 mg/kg, 40–60 mg/kg and 60–100 mg/kg. The results show that baicalin significantly reduced immobility time at doses of 40–60 mg/kg [SMD = −45.49, 95%CI (−60.44, −30.54), *P* < 0.00001] and 60–100 mg/kg [SMD = −28.57, 95%CI (−48.43, −8.71), *p* = 0.005], demonstrating a statistically significant difference. However, there was no statistical difference in TST with a baicalin dose of 20–40 mg/kg [SMD = −29.00, 95%CI (−62.50, 4.50), *p* = 0.09]. The results suggest that the dosage of baicalin has a significant impact on the TST. The subgroup analysis was conducted on the rats, and it was found that the immobility time in the TST was significantly shorter in the baicalin intervention group compared to the model group [SMD = −40.35, 95%CI (−51.70, −29.01), *P* < 0.00001], indicating that the rats do not affect the intervention effect of baicalin. Therefore, our results indicate that the dosage of baicalin significantly affects its efficacy in the TST. The optimal dosage of baicalin may be 40–60 mg/kg or 60–100 mg/kg. Additionally, different depression models and the use of rats do not significantly influence the improvement effect of baicalin on TST.

#### 3.7.3 FST

The animals were divided into two groups based on the animal model of depression: CUMS and CORT. The results showed that compared with the model group, the CUMS group [SMD = −44.78, 95%CI (−56.48, −33.09), *P* < 0.00001] and the CORT group [SMD = −30.16, 95%CI (−44.18, −16.14), *P* < 0.00001] both exhibited a significant reduction in immobility time after baicalin treatment. These findings indicate that baicalin’s improvement in FST is not affected by different animal models of depression. The baicalin intervention doses were also divided into four groups: ≤20 mg/kg, 20–40 mg/kg, 40–60 mg/kg and 60–100 mg/kg. Compared with the model group, the immobility time during the FST was significantly reduced in the baicalin group at doses of ≤20 mg/kg [SMD = −36.70, 95%CI (−53.47, −19.93), *P* < 0.00001], 20–40 mg/kg [SMD = −66.77, 95%CI (−88.77, −44.77), *P* < 0.00001], 40–60 mg/kg [SMD = −47.11, 95%CI (−60.22, −34.00), *P* < 0.00001], and 60–100 mg/kg [SMD = −17.21, 95%CI (−33.38, −1.04), *P* < 0.00001]. The results indicate that the improvement effect of baicalin on the FST is not affected by the dosage of baicalin. The animals were divided into two groups according to their species: rats and mouse. Compared with the model group, the immobility time during the FST was significantly reduced in both the rat [SMD = −48.98, 95%CI (−82.78, −15.18), *P* < 0.00001] and the mouse [SMD = −37.40, 95%CI (−46.53, −28.27), *P* < 0.00001] after treatment with baicalin, suggesting that the improvement in FST by baicalin is not affected by the species of the animals. Therefore, the improvement effect of baicalin on FST is not influenced by different depression models, baicalin doses, or animal species.

#### 3.7.4 OFT

In the animal model of depression, compared to the model group, baicalin significantly increased the frequency of crossing in the CUMS group [SMD = 32.44, 95%CI (17.74–47.13), *P* < 0.00001]. This suggests that the CUMS modeling method does not affect the efficacy of baicalin in improving performance in the OFT. The baicalin intervention doses were also divided into four groups: 20–40 mg/kg, 40–60 mg/kg and 60–100 mg/kg. The results show that baicalin significantly increased the frequency of crossing at doses of 40–60 mg/kg [SMD = 41.33, 95%CI (23.94–58.73), *P* < 0.00001] and 60–100 mg/kg [SMD = 27.00, 95%CI (12.63–41.37), *P* < 0.00001], demonstrating a statistically significant difference. However, there was no statistical difference with a baicalin dose of 20–40 mg/kg [SMD = 10.00, 95%CI (−6.36–26.36), *p* = 0.231]. The results suggest that the dosage of baicalin has a significant impact on the OFT. The animals were divided into two groups according to their species: rats and mouse. Compared with the model group, after baicalin treatment, the crossing frequency significantly increased in both the rat group [SMD = 18.97, 95%CI (2.33–35.60), *p* = 0.025] and the mouse group [SMD = 41.33, 95%CI (23.94–58.73), *P* < 0.00001], suggesting that the improvement in OFT by baicalin is not affected by the species of the animals. Therefore, our results indicated that the dosage of baicalin significantly affects its efficacy in the OFT. The optimal dosage of baicalin may be 40–60 mg/kg or 60–100 mg/kg. Additionally, different depression models and the animal species do not affect baicalin’s improvement in the OFT.

## 4 Discussion

Our meta-analysis included a total of 22 studies that assessed the efficacy of baicalin in treating depression through various behavioral tests. The main findings of this study are as follows: baicalin significantly improved the performance of depression animal models in the SPT, TST, FST, and OFT. Specifically, after treatment with baicalin, depressed animals exhibited a notable preference for sucrose, increased struggling time during suspension, increased vigorous activity during forced swimming, and a significantly higher frequency of exploration in the open field. These results collectively suggest that baicalin has a significant antidepressant effect. Subgroup analysis indicated that different doses of baicalin had varying effects on the behavioral improvement of depressed animals. At doses of 20–40 mg/kg, there was no significant improvement in the frequency of open field exploration or the struggling time during suspension. However, at doses of 40–100 mg/kg, baicalin significantly enhanced sucrose preference, exploration frequency, struggling time during suspension, and struggling time during forced swimming in depressed animals. Additionally, the choice of different depression models and animal strains did not seem to significantly affect the antidepressant effects of baicalin.

**FIGURE 9:**
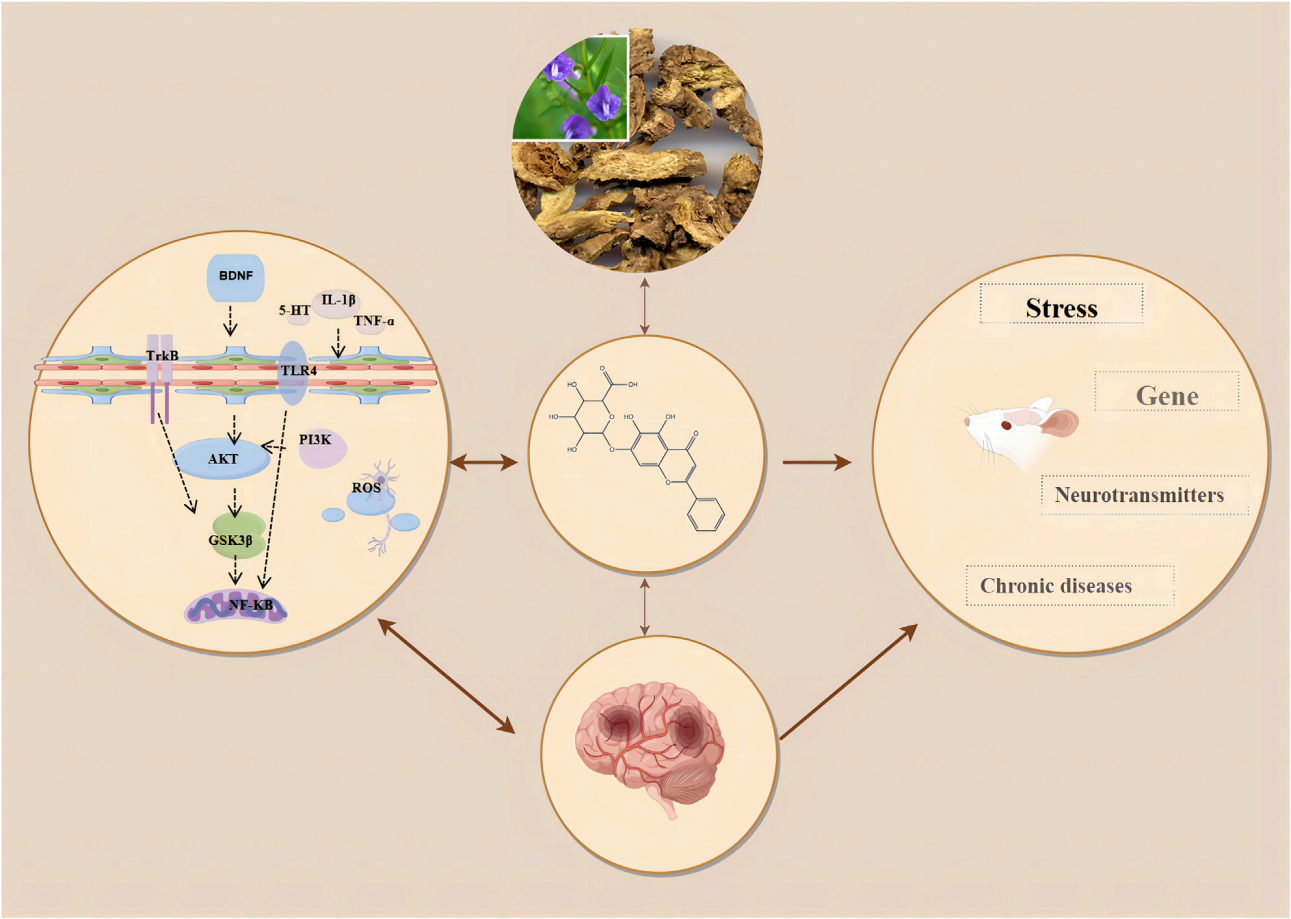
Mechanism of action of baicalin.

This study indicates that baicalin significantly improves the outcome measures of SPT, TST, FST, and OFT in animal models of depression. Our research findings are consistent with the systematic reviews by Ma and Wang et al. on the treatment of depression using Scutellaria baicalensis and its active components, although their studies did not explicitly demonstrate the efficacy of baicalin when used alone (Ma et al., 2024; Wang and Gao, 2024). Additionally, our results align with the effects of antidepressants fluoxetine and imipramine in reversing depressive behaviors in CUMS rats, further supporting the potential role of baicalin in depression treatment (Ossowska et al., 2004). The four outcome indicators of SPT, TST, FST, and OFT respectively reflect the different depressive states of patients. Specifically, the SPT indicates anhedonia, demonstrating a diminished capacity to anticipate or enjoy pleasurable activities. The TST and FST measure feelings of helplessness and despair, respectively. The OFT assesses increased anxiety. These indicators of anhedonia, despair, helplessness, and anxiety are critical for assessing depression progression and treatment response. Existing evidence has shown that baicalin improves symptoms in depression primarily through anti-inflammatory effects, inhibition of oxidative stress, regulation of the HPA axis, and restoration of neuroplasticity (Shiyuan et al., 2023). (Figure 9). It has been found that neuroinflammation and oxidative stress are closely related to anhedonia and anxiety in patients with depression (da Silva et al., 2024). Neuroinflammation can lead to the overactivation of microglia, which inhibits synaptic transmission in hippocampal neurons, causing neuronal dysfunction and resulting in depressive symptoms (Zou et al., 2022). Numerous studies have shown that inflammatory mediators and cytokines such as interleukin-1β (IL-1β), interleukin-6 (IL-6), and tumor necrosis factor-α (TNF-α) are frequently present in the peripheral blood of patients with depression (Tian et al., 2024). In patients with depression, pathogen-associated molecular patterns (PAMPs) such as inducible nitric oxide synthase (iNOS) and nitric oxide (NO), and danger-associated molecular patterns (DAMPs) such as IL-1β and interleukin-18 (IL-18) enter the cytoplasm of hippocampal microglia (Tian et al., 2024). These are recognized by the pattern recognition receptor NOD, LRR, and pyrin domain-containing protein 3 (NLRP3), stimulating an immune response (Mokhtari and Uludag, 2024). This leads to the recruitment of NLRP3, apoptosis-associated speck-like protein containing a CARD (ASC), and Pro-caspase-1 (Cysteine-aspartic protease-1) (Huang et al., 2023). Activated caspase-1 cleaves Pro-IL-1β and Pro-IL-18 into their active forms, IL-1β and IL-18, thereby stimulating hippocampal inflammatory cells, exacerbating inflammation, promoting the formation of neurotoxic A1 astrocytes, and inducing depressive-like behaviors such as anxiety and despair (Huang et al., 2023). Liu et al. found that baicalin significantly inhibited the levels of inflammatory factors in serum, hippocampal homogenate and PC12 cell medium in CUMS rat models (Liu and Liu, 2017). Furthermore, research has proved that baicalin significantly downregulates the expression of high mobility group box 1 (HMGB1), toll-like receptor 4 (TLR4), and phosphorylated nuclear factor kappa-light-chain-enhancer of activated B cells (NF-κB) p65 in the hippocampus of CUMS mice, and inhibits the levels of IL-1β, IL-6, and TNF-α in serum and hippocampal homogenate (Liu et al., 2019). These findings suggest that baicalin may regulate the HMGB1/TLR4/NF-κB pathway to exert anti-neuroinflammatory effects and improve depressive symptoms (Liu et al., 2019). There is evidence that HMGB1 is a key trigger in the crosstalk between normal and inflammatory cells. HMGB1 binds to TLR4, leading to the recruitment and promotion of NF-κB, thereby activating the transcription of inflammatory cytokines, chemokines, and adhesion molecules, and promoting the development of depression (Jia et al., 2024). Glycogen synthase kinase three beta (GSK3β) is a serine/threonine kinase widely present in the central nervous system, primarily localized in neurons (Olianas et al., 2011). When the activity of GSK3β increases, it can promote neuronal apoptosis, whereas decreased activity can regulate neuronal plasticity (Jin et al., 2019). The primary mechanism by which neurons regulate GSK3β activity is through phosphorylation at its Ser9 site (Ahmed et al., 2023). Studies have shown that the regulation of GSK3β activity is directly or indirectly related to the mechanisms of action of many mood stabilizers and antidepressants, primarily mediated through cAMP response element-binding protein (CREB), which regulates the transcription of BDNF to achieve an antidepressant effect (Lee et al., 2019). Zhang et al. found that baicalin might exert neuroprotective effects by inhibiting the activation of the GSK3β/NF-κB/NLRP3 signaling pathway, promoting neuronal maturation, and preventing neuronal apoptosis (Zhang C. Y. et al., 2018). Katrenčíková et al. discovered that antidepressant treatment significantly increased superoxide dismutase (SOD) activity in patients with depression. In recent years, studies have found that baicalin inhibits hippocampal neuronal apoptosis in CUMS rats by reducing malondialdehyde (MDA) levels and increasing superoxide dismutase (SOD) levels (Katrenčíková et al., 2021). Similarly, Fu et al. found that baicalin enhanced SOD activity, inhibited MDA production, downregulated NF-κB levels, and significantly increased nuclear nuclear factor erythroid 2-related factor 2 (Nrf2) content (Fu et al., 2021). Evidence suggests that the anxiety and despair symptoms in depression patients might originate from hyperactivity of the HPA axis, leading to the excessive release of stress hormones such as corticotropin-releasing hormone (CRH), adrenocorticotropic hormone (ACTH), and CORT, resulting in impaired and dysregulated negative feedback mechanisms in the body (de Kloet et al., 2005). High levels of stress hormones further damage the structure and function of the hippocampus, causing atrophy and dysfunction of pyramidal neurons, ultimately manifesting as depressive symptoms (Myers et al., 2014). Zhang et al. found that baicalin treatment restored glucocorticoid receptor (GR) protein levels in the cytoplasm and nucleus of CORT-treated mice, reduced corticosterone and ACTH levels, restored HPA axis negative feedback function, and alleviated depressive symptoms Zhang et al. (2016). Neuroplasticity refers to the reversible structural and functional changes in the brain under stress and other conditions (Minbay et al., 2024). Clinical studies have shown that patients with depression often exhibit reduced hippocampal volume, which is associated with decreased neurogenesis and impaired neuroplasticity (Medeiros et al., 2024). Gao et al. demonstrated that neuronal apoptosis in the damaged hippocampal region is associated with anxiety- and depression-like behaviors, and identified amyloid beta precursor protein-like 2 (APPL2) as a potential therapeutic target for depression and olfactory deficits (Gao et al., 2018). Baicalin inhibited the APPL2/GR signaling pathway in APPL2 transgenic mice and corticosterone-induced depressed mice, promoting neuronal apoptosis in the subventricular zone (SVZ), olfactory bulb (OB), and hippocampus of APPL2 transgenic mice (Gao et al., 2018). This suggests that the antidepressant mechanism of baicalin might be related to the regulation of the APPL2/GR signaling pathway. Zhang et al. demonstrated that baicalin activates protein kinase B (Akt) and upregulates forkhead box G1 (FOXG1) in the hippocampus, thereby helping prevent neuronal apoptosis (Zhang et al., 2019a). Recent studies have found that baicalin increases the protein expression of Wnt3a, dishevelled segment polarity protein 2 (DVL2), and β-catenin in the hippocampus, inhibits the phosphorylation level of GSK3β, thereby stabilizing β-catenin, which is critical for neurogenesis (Xiao et al., 2021). Meanwhile, Lu et al. discovered that baicalin activates Ras-related C3 botulinum toxin substrate 1 (Rac1) and enhances LIM domain kinase (LIMK) phosphorylation in the hippocampal CA3 region, which is crucial for dendritic spine formation and synaptic plasticity (Lu et al., 2019). The activation of cAMP promotes neurogenesis and synaptic plasticity, improving anhedonia (Chen et al., 2018). Zhang et al. pointed out that baicalin increases cAMP levels in the dentate gyrus of the hippocampus, activates protein kinase A (PKA), subsequently phosphorylates CREB, enhancing its transcriptional activity, thereby regulating mood and cognitive functions (Zhang et al., 2018b). In summary, baicalin on inflammatory responses, oxidative stress, regulation of the HPA axis and modulation of neuroplasticity via HMGB1/TLR4/NF-κB, PI3K/Akt/FoxO1, GSK3β/NF-κB/NLRP3, TLR4/NF-κB/NLRP3, Akt/FOXG1, Wnt/β-catenin, Rac1/LIMK/Cofilin, and cAMP/PKA pathways.

### 4.1 Study implications for research and practice

The management of depression has become a significant global public health issue (Mohamed Ibrahim et al., 2020). Currently, the single-drug efficacy of commonly used antidepressants like SSRIs and SNRIs is often inadequate, leading to patient disappointment in treatment outcomes (Fagiolini et al., 2021). Clinicians may attempt dose escalation or combination therapies to improve efficacy, which can lead to unnecessary side effects, particularly affecting cardiovascular, nervous, or mental health (e.g., increased suicide risk, serotonin syndrome, arrhythmias, and liver damage) (Liu et al., 2017; Papakostas et al., 2018). Therefore, there is an urgent need to develop new antidepressant drugs that can achieve significant therapeutic effects in the short term with fewer side effects. Baicalein, a traditional herbal medicine, offers multitarget properties, accessibility, and high safety compared to conventional Western medications (Munjal et al., 2024). However, no Chinese herbal extracts have been marketed for formal promotion as antidepressant drugs. The research on baicalin in treating depression remains at the stage of animal experiments. To advance baicalin as a novel drug for clinical depression treatment, it is crucial to validate its efficacy in animal models. Thus, this article aims to comprehensively assess and meta-analyze the efficacy of baicalin in depression animal models to clarify its potential value in depression treatment and promote its further application in clinical practice.

## 5 Conclusion

The results of this systematic review support the therapeutic effects of baicalin on animal models of depression. This is evidenced by a significant increase in sucrose preference, a notable decrease in immobility time, and a marked increase in exploration frequency after baicalin treatment. Additionally, the antidepressant effects of baicalin may be dose-dependent, with optimal efficacy observed at daily doses of 40–100 mg/kg. This study provides an evidence-based evaluation for the development and utilization of baicalin. Therefore, we suggest that baicalin may be a promising candidate for the treatment of depression, warranting further experimental and clinical trials.

## 6 Limitation

Firstly, 22 studies did not employ blinded assessments, which may compromise the validity of the results. Secondly, there was no uniform standard across different studies concerning animal interventions, drug dosage, treatment protocols, and model species, contributing to high heterogeneity. Although we employed subgroup analysis to further elucidate the findings, higher-quality randomized controlled trials are still needed in the future to provide reliable evidence to validate the results of animal experiments and to better clarify the antidepressant effects of baicalin.

## Data Availability

The original contributions presented in the study are included in the article/Supplementary Material, further inquiries can be directed to the corresponding author.
